# What is the impact of stress on the onset and anti-thyroid drug therapy in patients with graves’ disease: a systematic review and meta-analysis

**DOI:** 10.1186/s12902-023-01450-y

**Published:** 2023-09-12

**Authors:** Jing Wang, Zhichao Chen, Ciriaco Carru, Giampiero Capobianco, Stefania Sedda, Zhi Li

**Affiliations:** 1 Department of Obstetrics and Gynecology, Second Affiliated Hospital of Shantou University Medical College, Shantou, China; 2 Department of Biomedical Sciences, University of Sassari, Sassari, Italy; 3 Department of Cardiology, Second Affiliated Hospital of Shantou University Medical College, Shantou, China; 4 Department of Medical, Surgical and Experimental Sciences, University of Sassari, Sassari, Italy

**Keywords:** Stress, Stressful life events, Graves’ disease, Thyroid gland, Meta-analysis

## Abstract

**Background:**

The effect of stress on Graves’ disease (GD) is controversial. Our purpose was to quantify the impacts of stress on patients with Graves’ disease.

**Methods:**

Systematic searches of PubMed, MEDLINE, Embase, Web of Science, Scopus, Cochrane Library and PsycInfo were conducted from inception to 1 January 2023. Studies comparing the incidence of stressful life events (SLEs) that occurred before diagnosis and during drug therapy in cases diagnosed with GD and controls were included in the final analysis.

**Results:**

Nine case-control studies and four cohort studies enrolling 2892 participants (1685 [58%] patients) were included. Meta-analysis revealed a high and significant effect-size index in a random effect model (d = 1.81, *P* = 0.01), indicating that stress is an important factor in the onset of GD. The relationship between SLEs and GD was stronger in studies with higher proportions of female patients (β = 0.22, P < 0.01) and weaker in studies with older patients with GD (β =−0.62, *P* < 0.01). However, stress did not significantly affect the outcome of antithyroid drug therapy for GD (d = 0.32, *P* = 0.09).

**Conclusions:**

The results of this meta-analysis suggest that stress is one of the environmental triggers for the onset of GD. Therefore, we recommend stress management assistance for individuals genetically susceptible to GD, especially for young females.

**Supplementary Information:**

The online version contains supplementary material available at 10.1186/s12902-023-01450-y.

## Background

Graves’ disease (GD) is the most common etiology of hyperthyroidism, affecting approximately 0.2% of males and 2% of females worldwide (with a male-to-female ratio of 1: 5 ~ 10) [1]. Stress is the complex psychophysiological response of the body when homeostasis or the internal environment’s steady state is disturbed or imperilled [2]. Stress can directly impact health via neuroendocrine and autonomic responses, but it also indirectly affects health by changing a healthy lifestyle. The role of stress in developing Graves’ disease was hypothesized very early on, particularly during wartime. A prospective cohort study conducted during the civil war in Serbia (former Yugoslavia) reveals that the incidence of Graves’ disease dramatically increased from 1992 to 1995 [3]. Therefore, physicians are often aware of the role of stress in causing the disease and the efficacy of the treatment in clinical practice [4].

The development of GD is tightly linked to genetic and environmental factors. Individuals carrying susceptibility genes, triggered by certain environmental factors, initiate the process of autoimmune pathogenesis [5]. Environmental factors such as smoking, dietary iodine, infections, pregnancy and emotional stress are considered potential triggers for Graves’ disease [6]. How these different factors interact to produce GD risk has yet to be entirely clarified. However, a growing number of studies in animal and human models have found that chronic activation of stress responses leads to the overproduction of catecholamines and glucocorticoids, which suppress the immune response [7–9].

“Anxiety”, “emotional instability”, “insomnia”, “irritability”, “sensitivity” and “depression”, etc. are general mental symptoms of GD patients [10]. Those studies investigating mental symptoms and GD are unable to come up with a convincing causal relationship. Thus, Winsa et al. [11] quantified stressful emotions into measurable stressful life events and reported a case-control study of stressful life events (SLEs) occurring in the 12 months before the diagnosis of GD patients versus a healthy population. Over 2 years, 208 (95%) of 219 eligible GD patients claimed to have had SLEs in the 12 months before the diagnosis compared with controls. After this report, several case-control studies also explored the association between SLEs and the onset of GD [12–14]. Conversely, some studies did not find the same association between onset and stressful life events in patients diagnosed with GD [15]. Therefore, the role of stress needs further evaluation.

Our study aims to analyze the association between stress and Graves’ disease to provide a clear view.

## Materials and methods

### Registration

The systematic review and meta-analysis protocol was registered on PROSPERO (ID: CRD42023389041). Our research was performed according to the Preferred Reporting Items for Systematic Reviews and Meta-Analyses (PRISMA) protocols. The PRISMA checklist is available in the supplementary material (Supplementary Table S1).

### Data sources and searches

A systematic search was conducted in the following electronic databases: PubMed, Embase, MEDLINE, Scopus, Web of Science, Cochrane Library and PsycInfo from inception to 1 January 2023. The keywords we used for searching were “stressful life events,” “psychosocial stress,” “life stress,” “emotional stress,” “mental stress,” “stress,” “hyperthyroidism,”“Graves Disease,” “cohort studies,” “cohort studies,” “cross-sectional studies,” and “case-control studies”. We also conducted a manual search in the references of included articles to obtain additional records.

### Inclusion and exclusion criteria

The included studies satisfied the following criteria:

- Included patients had clinical and laboratory confirmation of Graves’ disease diagnosis.
- Documented assessment of stressful life events or scores.
- To compare the incidence of SLEs before the diagnosis of Graves’ disease, populations included patients newly diagnosed with GD and healthy controls.
- To compare the incidence of SLEs after at least 12 months of antithyroid drug therapy, populations included the noncured GD group and the cured GD group.
- Published in English with accessible publications.

Studies were excluded if they (1) were not specifically referred to Graves’ disease; (2) lacked a comparison group; or (3) were reviews, comments and conference abstracts.

### Study selection

Two investigators (JW, ZC) reviewed study titles, abstracts and full texts independently to confirm eligibility. Two investigators were in charge of the data extraction, quality assessment and detailed analysis of the included studies. Any disagreement between the investigators was discussed, and an agreement was reached with a third independent investigator (CC).

### Data extraction

Two investigators piloted a data table to extract the following data from the included studies independently: authors, country, year of publication, sample size, study design, mean age of participants, proportion of females in the study population, diagnostic criteria for GD, tools for assessing stress life events, mean, standard deviation of SLE scores and data in the study and the outcomes. If the interquartile or range were reported in the studies, those data were transformed into mean (standard difference) [17, 18]. For those data only presented in graphs, we used WebPlotDigitizer (Author: Ankit R, Website: https://automeris.io/WebPlotDigitizer, Version: 4.6, Date: 10th January 2023, Location: California, USA) to extract the data from the figures.

### Quality assessment

Two of us (JW, ZC) independently assessed the quality of each study with the Newcastle-Ottawa Quality Assessment Scale (NOS) [19]. The validity of NOS has been established based on a critical review of the items by experts to assess the quality of studies to be used in a meta-analysis. It was developed to address the quality of nonrandomised studies (i.e. case-control and cohort studies) with its content, design and ease of use. A ‘star system’ has been devised that evaluates a study based on three broad criteria: the selection of the study groups (4 stars); the comparability among the groups (2 stars); and the identification of the exposure or outcome of interest for cohort or case-control studies (3 stars), respectively. Studies with a score of 9 were considered high quality, while those with a score of 7–8 were medium quality and those below 7 were low quality. Disagreements were settled through discussion to reach a consensus.

### Statistical analysis

Standardized mean differences (SMDs) and 95% confidence intervals (95% CI) were calculated for every study that assessed the score or number of SLEs compared to controls in patients with GD. P values < 0.05 were considered statistically significant in all analyses.

Cohen’s formula for the standardized mean difference calculation:

1 $${\text{d = }}\left( {{{\text{X}}_{{\text{SLE GD}}}}{\text{-- }}{{\text{X}}_{{\text{SLE CONTROL}}}}} \right){\text{/S}}{{\text{D}}_{{\text{Pooled}}}}$$

X_SLE GD_ and X_SLE CONTROL_ are the means of SLEs or scores in the patients with GD and control groups, respectively, and SD_Pooled_ is the pooled standard deviation. In the Cohen model, effect sizes were classified as high ≧ 0.8, moderate = 0.5 and small = 0.2 [20].

The heterogeneity of pooled effect sizes was evaluated using the Q test and the values of I^2^ statistics. *I*^2^ = 0–50% (low to moderate heterogeneity), while *I*^2^ above 50% was considered medium to high statistical heterogeneity [21]. Because there was high heterogeneity in the effect sizes calculated from the studies included, random-effects model meta-analyses were performed. Otherwise, we applied the fixed model for calculation. Meta-regression and stratified analyses included the proportion of female sample, mean sample age, NOS scores, location and tools for stressful events assessment. Sensitivity analysis was performed by sequentially removing one study at a time to examine the internal consistency of the results. The purpose was to verify the stability of our study results after excluding the effect of individual studies. We assessed potential publication bias by the Egger weighted regression test [22]. All statistical analyses were performed using R statistical software version 4.2.1.

## Results

### Study selection

**Fig. 1:**
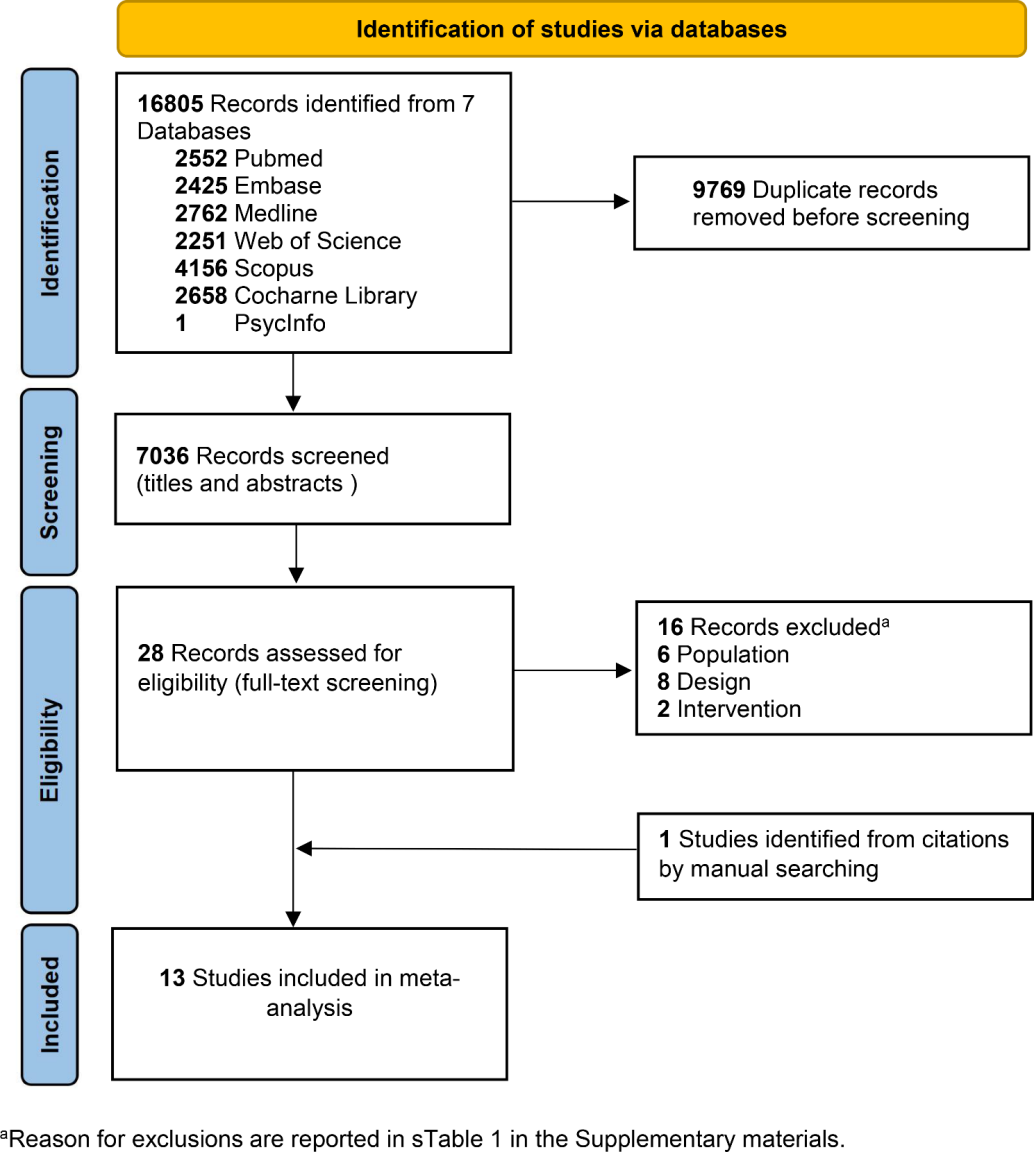
Search flow diagram

Figure 1 summarizes the literature selection process. A total of 16,805 records were identified according to our search strategy from 7 databases. Twenty-eight studies were relevant for a full-text review and 16 studies were excluded due to the wrong study design, wrong population, absence of a control group and absence of SLE assessment. One study was identified from citations by manual searching. Finally, a total of 13 studies met the inclusion criteria and were included in the final analyses. The reasons for exclusion are shown in the supplementary material (Supplementary Table S2).

### Characteristics of the included studies

Thirteen studies enrolling 2892 subjects (1685 [58%] patients with GD and 1207 [42%] controls) from 9 countries were included. Among the thirteen studies, nine studies compared the effect of SLEs on the onset of GD and the other four studies compared the effect of SLEs on the efficacy of medications for GD. The mean age of the subjects was 38.5 years, and most of them were women (2372 [82%] vs. 520 [18%] men). Four studies applied semistructured interviews and nine studies applied self-rating questionnaires for stressful event assessment. Four instruments were used across studies to evaluate stressful life events, including the Life Experiences Survey (LES), Paykel’s Interview for Recent Life Events (PIRLE), the Holmes and Rahe Life Events Scale (HRLES), and Natsume’s Stress Inventory (NSI). Table 1 and Supplementary Table S3 summarizes the descriptive characteristics of the included studies.

**Table 1:** Selected Characteristics of 13 studies included in meta-analysis

Source	Country	Sample size, (cases/noncases)	Age (mean), y	Female, %	Tools for stressful events	
Gray et al., 1985 [15]	UK	50/50	43.1	76.0	Semi-structured interview	
Winsa et al., 1991 [11]	Sweden	208/372	40.2	82.0	Self-rating questionnaires (LES)	
Sonino et al., 1993 [12]	Italy	70/70	39.2	82.9	Semi-structured interview (PIRLE)	
Kung et al., 1995 [13]	China	95/95	33.1	84.2	Self-rating questionnaires (LES)	
Radosavljević et al., 1996 [14]	Serbia	100/100	40.6	93	Semi-structured interview (PIRLE)	
Yoshiuchi et al., 1998 [16]	Japan	228/228	38.4	79.8	Self-rating questionnaires (HRLES)	
Matos-Santos et al., 2001 [23]	Portugal	31/31	38.4	71	Self-rating questionnaires (LES)	
Pintor et al., 2003 [24]	Philippines	224/224	37.1	86.6	Self-rating questionnaires (LES)	
Topcu et al., 2012 [25]	Turkey	45/37	40.9	73.3	Self-rating questionnaires (LES)	
Yoshichi et al., 1998 [26]	Japan	155/75^a^	38.4	79.1	Self-rating questionnaires (HRLES)	
Fukao et al., 2003 [27]	Japan	41/28^a^	41.0	94.2	Self-rating questionnaires (NSI)	
Chen et al., 2012 [28]	China	129/148^a^	37.9	72.9	Self-rating questionnaires (LES)	
Vita et al., 2014 [29]	Italy	43/15^a^	35.4	62.1	Semi-structured interview	
**Study design**	**Outcome**	**Diagnosis**				**Quality**
Case-control	GD	Chemical tests, diagnosis by physician				7
Case-control	GD	Clinical signs, raised thyroid hormone, suppressed TSH, thyroid-related antibodies				9
Case-control	GD	Clinical features, increased thyroxine, suppressed TSH, thyroid-related antibodies				8
Case-control	GD	Clinical features, thyroid scan, increased thyroxine, suppressed TSH				7
Case-control	GD	Clinical signs, increased thyroxine, suppressed TSH, thyroid scintigraphy				8
Case-control	GD	Clinical features, increased thyroxine, decreased TSH, thyroid scintigraphy, thyroid-related antibodies				7
Case-control	GD	Clinical criteria, increased thyroxine, suppressed TSH, thyroid-related antibodies				8
Case-control	GD	Clinical signs, raised thyroxine, decreased TSH, thyroid-related antibodies				6
Case-control	GD	Thyroxine, TSH suppression, thyroid-related antibodies, thyroid USG				7
Prospective cohort study	Drug efficacy	Concentrations of FT4, FT3, TSH, and TBII				7
Prospective cohort study	Drug efficacy	serum-free thyroxine,TSH concentrations				8
Prospective cohort study	Drug efficacy	Thyroxine, TSH concentrations				8
Prospective cohort study	Drug efficacy	suppressed levels of TSH, levels of FT3 and/or FT4.				9

Abbreviations: LES: The Life Experiences Survey; PIRLE: Paykel’s Interview for Recent Life Events; HRLES: the Holmes and Rahe life events scale; NSI: the Natsume’s Stress Inventory; GD: Graves’ disease; TSH: Thyroid stimulating hormone; USG: Ultrasonography; FT: Serum free thyroxine; TBII: thyroid binding inhibitory immunoglobulins; ^a^ cured cases/not-cured cased or recurrences cases;

### Risk of bias

Under the assessment of NOS, one study scored a 9 (high quality), six studies obtained a score of 8 (medium), four of 7 (medium), and the remaining one scored 6 (low quality). Among case-control studies, five (55.6%) showed control selection bias, with hospital controls recruited instead of community controls. Two studies (22.2%) did not specifically describe the effect of any additional factors, such as gender, age, and education. Four studies (44.4%) did not report the response rate. Among the cohort studies included, three (75%) had a potential bias in the assessment of outcomes. One study reported a low follow-up rate of 75% and no detailed description of those lost. Table 2 summarizes the quality assessment of various studies using the Newcastle-Ottawa Quality Assessment Scale.

**Table 2:** Qualities of studies included in meta-analysis

Study name	Selection of subjects				Comparability		Exposure			
	Q1	Q2	Q3	Q4	Q5	Q6	Q7	Q8	Q9	Score
Gray et al.	Y	Y	N	Y	Y	N	Y	Y	Y	7
Winsa et al.	Y	Y	Y	Y	Y	Y	Y	Y	Y	9
Sonino et al.	Y	Y	Y	Y	Y	Y	Y	Y	N	8
Kung et al.	Y	Y	Y	Y	Y	Y	N	Y	N	7
Radosavljević et al.	Y	Y	N	Y	Y	Y	Y	Y	Y	8
Yoshiuchi et al.	Y	Y	N	Y	Y	Y	Y	Y	N	7
Matos-Santos et al.	Y	Y	Y	Y	Y	N	Y	Y	Y	8
Pintor et al.	Y	Y	N	Y	N	Y	Y	Y	N	6
Topcu et al.	Y	Y	N	Y	Y	N	Y	Y	Y	7
	**Selection**				**Comparability**		**Outcome**			
	**Q1** ^*^	**Q2**	**Q3**	**Q4**	**Q5**	**Q6**	**Q7**	**Q8**	**Q9**	
Yoshichi et al.	Y	Y	Y	Y	Y	Y	N	Y	N	7
Fukao et al.	Y	Y	Y	Y	Y	Y	N	Y	Y	8
Chen et al.	Y	Y	Y	Y	Y	Y	N	Y	Y	8
Vita et al.	Y	Y	Y	Y	Y	Y	Y	Y	Y	9

Q1-Q9: Question 1 to question 9 used to assessment the quality of case control studies in the Newcastle - Ottawa Quality Assessment Scale.

Q1. Is the case definition adequate? Q2. Representativeness of the cases; Q3. Selection of Controls; Q4. Definition of Controls?

Q5. Were study controls selected as the most important factor? Q6. Were any additional factors for study controls? Q7. Ascertainment of exposure;

Q8. Was same method of ascertainment for cases and controls ? Q9. Non-Response rate;

Q1^*^-Q9: Question 1 to question 9 used to assessment the quality of cohort studies in the Newcastle - Ottawa Quality Assessment Scale.

Q1. Representativeness of the exposed cohort; Q2. Selection of the non-exposed cohort; Q3. Ascertainment of exposure;

Q4. Demonstration that outcome of interest was not present at start of study; Q5. controls for age, sex and marital status; Q6. Study controls for other factors;

Q7. Assessment of outcome Q8. Was follow-up long enough for outcomes to occur? Q9. Adequacy of follow-up of cohorts;

Y, yes; N, no;

### Stress and the onset of graves’ disease

**Fig. 2:**
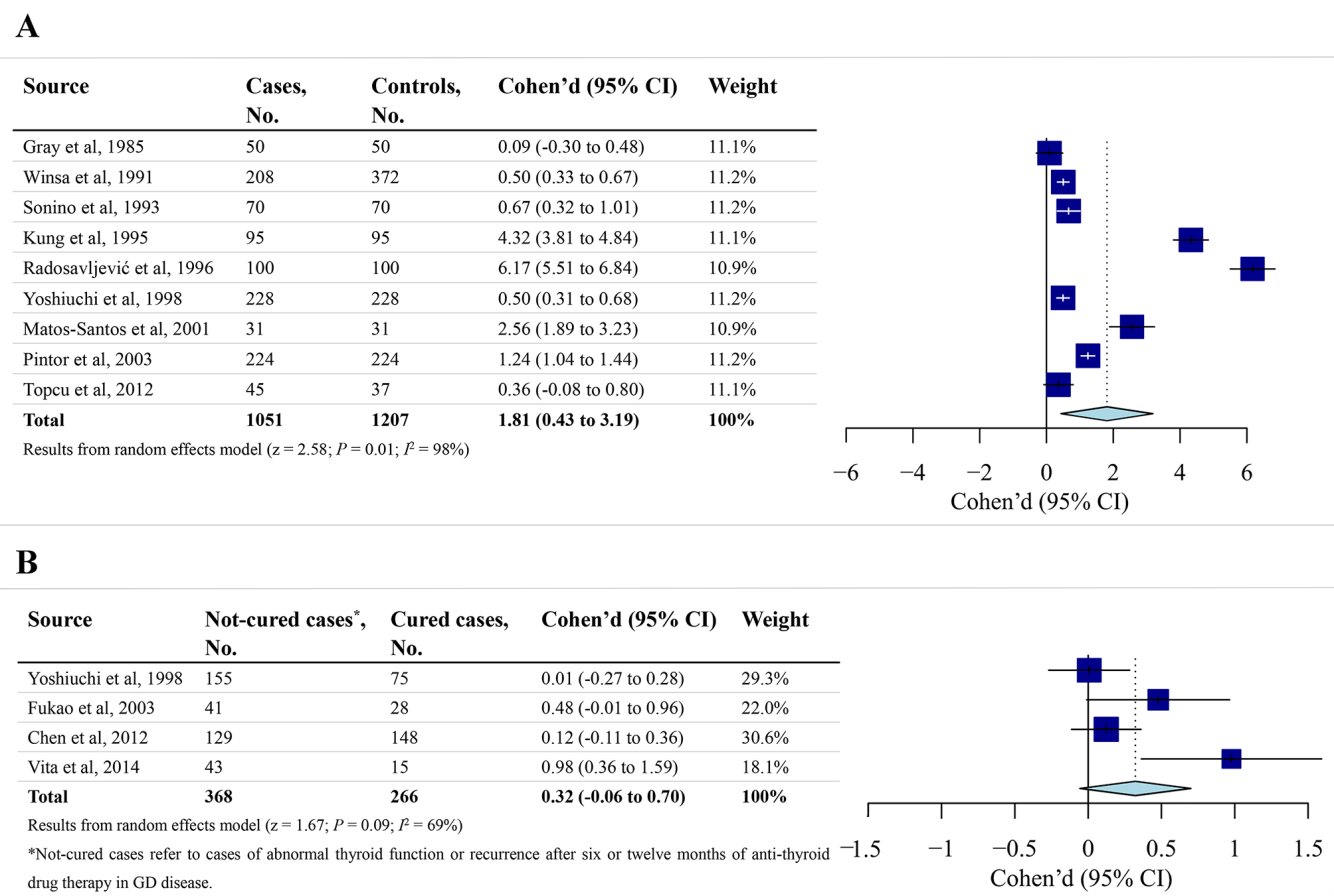
** A.** Forest plots of meta-analysis on the effect of SLEs in patients with GD before diagnosis. **B.** Forest plots of meta-analysis on the effect of SLEs in anti-thyroid drug therapy in patients with GD.

The analysis included 1051 patients newly diagnosed with GD and 1207 healthy controls from 9 studies. There was a significantly larger mean effect-size index for SLEs between GD patients and healthy controls (d = 1.81, 95% CI [0.43 to 3.19], Z-test = 2.58, *P* = 0.01), suggesting that Graves’ disease is associated with a significantly higher number of SLEs before diagnosis (Fig. 2A). Because the Q-test showed a significantly high heterogeneity (*Q* = 505, *I*^2^ = 98%, *P* < 0.001), a random-effects model was carried out for the analysis. In addition, meta-regression and stratified analyses were performed to determine the source of heterogeneity, including the proportion of female sample, mean sample age, NOS scores, location and tools for stressful event assessment.

### Meta-regression

The following meta-regression surveyed the role of gender and mean age of subjects as potential influencing factors of the relationship between SLEs and GD. The proportion of the female sample showed a positive association with the pooled effect-size index (β = 0.22, k = 9, 95% [0.07 to 0.36], *P* < 0.01), suggesting that the association between stress and the onset of GD was more substantial in studies with a large proportion of women.

**Fig. 3:**
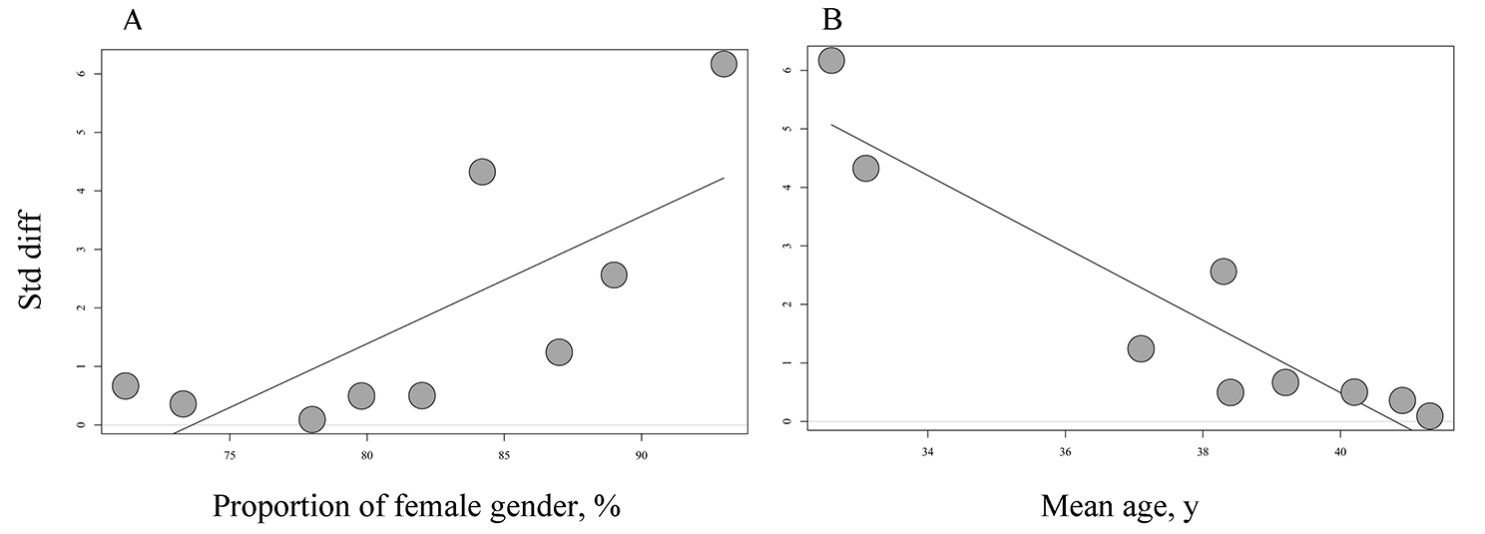
** A.** Meta-regression: sex at SLE testing in in patients with GD. **B.** Meta-regression: mean age at SLEs testing in in patients with GD.

The mean age of participants was also assessed as a potential influencing factor in the meta-regression analysis. The relation between stress and the onset of GD was weaker in studies that recruited older participants (β = −0.62, k = 9, 95% [−0.80 to−0.43], *P* < 0.001), suggesting that stress had a greater impact on the onset of GD in younger age groups (Fig. 3).

### Subgroup analysis

We conducted stratified analyses by the following factors: NOS scores (6–7, 8–9 points), location (Europe, Asia) and tools for SLE assessment (self-rating questionnaires, semi-structured interviews). The pooled effect size was larger in high NOS score studies (d = 2.46, k = 4, 95% [0.12 to 5.04], *I*^2^ = 99%) than in low-medium NOS score studies (d = 1.30, k = 5, 95% [−0.22 to 2.81], *I*^2^ = 98.2%), but the result was not significantly different (*Q* = 0.58, *P* = 0.44). The same statistics were applied to conduct the comparison based on location (*Q* = 0.04, *P* = 0.84) and tools for SLEs (*Q* = 0.13, *P* = 0.72). The results were not statistically significantly different.

Table 3 summarizes the pooled effect sizes for all the results, analysis of the relation between stressful life events and Graves’ Disease, meta-regression (moderating effects of female proportion and mean age in the study population) and subgroup analysis.

**Table 3:** Summary of meta-regression and stratified analysis of stressful life events and Graves’ disease

	k	d (*P* value)	β (*P* value)	95% CI	*I*^2^, %	Q (*P* value)
Comparison of stressful life events between patients with	9	1.81 (0.01)		0.43 to 3.19	98.0	
GD and control groups						
Meta-regression of female gender on relation between	9		0.22 (< 0.01)	0.07 to 0.36		
stressful life events and GD						
Meta-regression of mean age on relation between	9		−0.62 (< 0.01)	−0.80 to−0.43		
stressful life events and GD						
Stratified analysis by NOS score						0.58 (0.44)
Low-medium (6–7)	5	1.30 (0.09)		−0.22 to 2.81	98.2	
Medium-high (8–9)	4	2.46 (0.02)		0.12 to 5.04	99.0	
Stratified analysis by Location						0.04 (0.84)
Europe	6	1.71 (0.01)		0.16 to 3.58	98.4	
Asia	3	2.01 (0.09)		−0.28 to 4.30	98.9	
Stratified analysis by different assessment for						0.13 (0.72)
stressful life events						
Semi-structured interviews	3	2.30 (0.23)		−1.49 to 6.09	99.2	
Self-rating questionnaires	6	1.57 (0.01)		0.31 to 2.82	98.0	
Comparison of stressful life events between not-cured	4	0.32 (0.09)		−0.06 to 0.70	69.0	
patients and cured patients with GD after druy therapy						

### Sensitivity analysis and publication bias

**Fig. 4:**
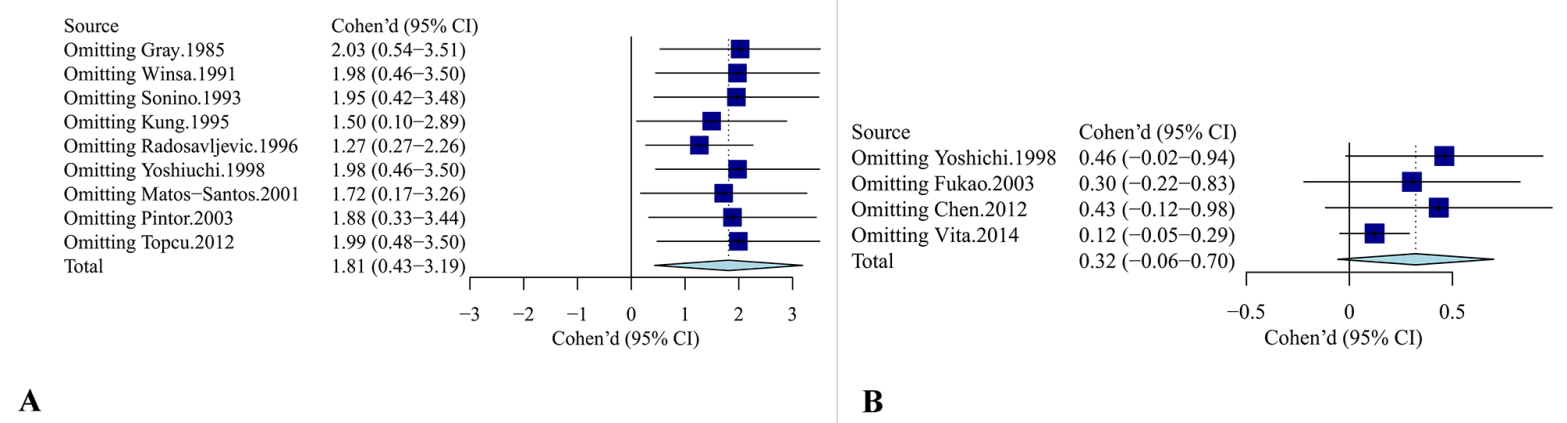
** A.** Sensitivity analysis on the effect of SLEs in patients with GD before diagnosis: based on a random effect model. **B.** Sensitivity analysis on the effect of SLEs in anti-thyroid drug therapy in patients with GD: based on a random effect model

We conducted a sensitivity analysis using an omit-one-out method to estimate potential sources of heterogeneity across the studies included in our study. This method suggested that the pooled effect sizes of stressful life events among studies included in our analysis remained stable and consistent. The pooled effect sizes of SLEs among studies varied from 1.27 [95%CI 0.27 to 2.26] to 2.03 [95% CI 0.54 to 3.51]. Figure 4A demonstrates the details of the sensitivity analysis.

**Fig. 5:**
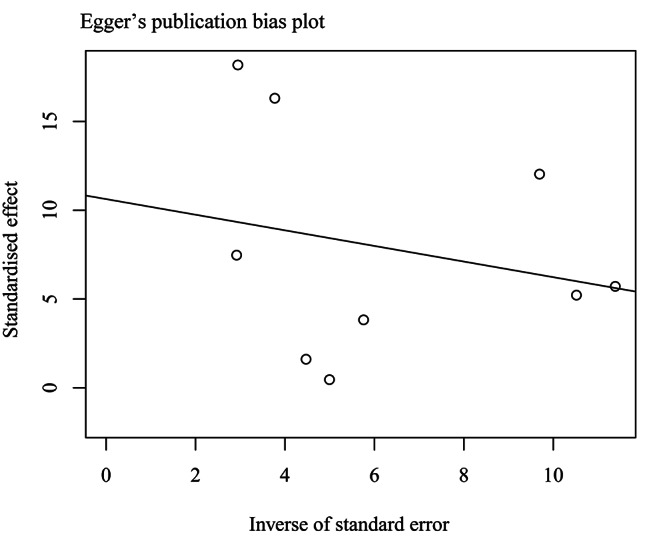
Egger’s publication bias plot for the association between SLEs and the onset of Graves’ disease

Figure 5 demonstrates the publication bias plot generated by Egger’s test. The plot shape and the test show a statistically non-significant result for publication bias (Intercept =−0.44, SE = 4.85, t = 2.19, *P* = 0.07). Overall, no potential publication bias was detected.

### Stress and the anti-thyroid drug therapy for graves’ disease

The four prospective cohort studies recruited 634 patients with GD, including 368 noncured cases and 266 cured cases. There was a larger effect size for stressful life events between noncured cases and cured cases but the result was not statistically significantly different (d = 0.32, 95% CI [−0.06 to 0.70], *I*^2^ = 69%, *P* = 0.09) (Fig. 2B). Sensitivity analysis showed the results remained stable and consistent by the omit-one-out method (Fig. 4B). Taken together, the results suggested no significant association between SLEs and the efficacy of antithyroid treatment in patients with GD.

## Discussion

### Main findings

To our knowledge, this study is the first systematic quantitative assessment of stressful life events and the onset of Graves’ disease and drug efficacy. Our review has assembled data from 13 studies involving 2892 subjects (1685 [58%] patients) in nine countries. Based on current evidence, our findings suggest that stressful life events are associated with the onset of Graves’ disease in individuals with genetic susceptibility to GD, suggesting that stress plays a significant role in the pathogenesis of GD. Meanwhile, we found moderating effects for gender and age in the relationship between SLEs and GD. The results revealed that in samples recruiting more female patients and younger patients, the relationship between SLEs and GD became stronger. However, whether stress is a risk factor in the efficacy of drug therapy in patients with GD needs further research.

### Stress and graves’ disease

Overall, the present analysis suggests a surprisingly high correlation between stressful life events and the onset of Graves’ disease (d = 1.81; 95% CI, 0.43 to 3.19). The high heterogeneity across studies led us to discover the moderating role of gender and age in stressors and the onset of GD. The proportion of the female sample showed a positive association with the pooled effect size (β = 0.22; 95% CI, 0.07 to 0.36) while the age of patients negatively regulated the association (β = −0.62; 95% CI, −0.80 to−0.43). Unexpectedly, stressful life events were not significantly associated with drug efficacy outcomes in patients with GD (d = 0.32; 95% CI, −0.06 to 0.70).

### Comparison, explanation and connection

Previous literature reviews have reported the relationship between mental disorders and Graves’ disease, including anxiety, depression and stress [30]. However, our study further quantified the association between the number of stressful life events or scores and the onset of disease in patients with GD. We excluded 3 studies that recruited not only patients with Graves’ disease but also patients with other types of hyperthyroidism and Graves’ ophthalmopathy. We also excluded 4 studies that lacked a control group or with non-healthy controls. Therefore, our study attempted to isolate the actual association between stress and the onset of Graves’ disease and drug efficacy.

It is well known that the human stress system is composed of the autonomic nervous system (ANS) and the hypothalamic-pituitary-adrenal (HPA) axis [31]. Many studies have indicated that various psychosocial factors such as stressful life events, trauma and distress in daily life become potent and chronic stressors that disrupt the stress system in the human body [32–34]. Stressors activate the HPA axis, which is associated with reduced production of thyrotropin (TSH). Thus, it inhibited the conversion of relatively inactive thyroxine (T4) to active triiodothyronine (T3) in peripheral tissues [35]. Graves’ disease has long been considered a predominantly T helper 2 (Th2) autoimmune disorder [36]. The imbalance and increased differentiation of Th2 may contribute directly to the onset of GD. During stress stimulation, glucocorticoids and catecholamines are released from the adrenal glands and locus coeruleus [37]. Glucocorticoids inhibit the production of Interleukin 12 (IL12) by antigen-presenting cells (APCs) and decrease IL12 receptor expression on T cells. Conversely, they increase the production of IL4 and IL10 by Th2 cells, resulting in an imbalance in favor of Th2 cells and the emergence of humoral immunity [38]. Catecholamines exert a comparable effect. Other mechanisms also connect stress to GD besides immune system stimulation by glucocorticoids and catecholamines. Stress is characterized by generating proinflammatory cytokines, including IL6, a cytokine produced by T cells and macrophages. Stress-induced increases in serum IL6 levels directly cause the Th1/Th17/Treg imbalance implicated in autoimmune disorders [39]. As these neurohumoral immunity, hormones and cytokines regulation mechanisms, stress has an impact on GD development in multiple pathways.

### Limitations

Several limitations in our meta-analysis should be emphasized. First, we did not include non-English publications or ongoing studies. Second, the heterogeneity among the studies should be noted in the interpretation of results. Although we conducted subgroup analysis and meta-regression to explore the sources of heterogeneity, the possible cause of heterogeneity could be differences in stressors across studies or in the timing of stressful events. Third, although validated life stress event scales or semi-structured interviews were applied in all included studies, there could be a risk of recall bias in reporting SLEs.

### The impacts of the findings

Our study has the following clinical impacts: (1) We suggest that future studies should be designed into prospective cohort studies to provide stronger evidence for the current findings. (2) We recommend stress management assistance for individuals genetically susceptible to Graves’ disease, especially young females. (3) Based on the current evidence, stressful life events were not associated with poor outcomes of drug therapy in patients with Graves’ disease. However, multicenter, large trials are warranted to draw a definitive conclusion.

## Conclusion

Our study indicated that patients with Graves’ disease experience more stressful life events before the diagnosis of the disease, suggesting that stress is one of the environmental triggers. This association is vital, especially in the young female population. Social and medical care is necessary to provide stress management and support for individuals with high genetic susceptibility to Graves’ disease.

To date, we cannot conclude a relationship between stress and drug efficacy in patients with Graves’ disease. More studies are required in the future to bring us to a definitive conclusion.

### Electronic supplementary material

Below is the link to the electronic supplementary material.

Supplementary Material 1

Supplementary Material 2

Supplementary Material 3

## Data Availability

All data generated or analyzed are in the text and supplementary materials.

## Abbreviations

GD: Graves’ disease
SLEs: Stressful life events
GO: Ophthalmopathy
NOS: Newcastle-Ottawa Quality Assessment Scale
LES: Life Experiences Survey
PIRLE: Paykel’s Interview for Recent Life Events
HRLES: Holmes and Rahe life events scale
NSI: Natsume’s Stress Inventory
SMD: Standardized mean difference
ANS: Autonomic nervous system
HPA: Hypothalamic-pituitary-adrenal
TSH: Thyrotropin
T4: Thyroxine
T3: Triiodothyronine
