# Supplementary material for: What is the impact of stress on the onset and anti-thyroid drug therapy in patients with graves’ disease: a systematic review and meta-analysis
Source: BMC Endocr Disord. 2023 Sep 12;23:194. doi: 10.1186/s12902-023-01450-y (PMC10496195; doi:10.1186/s12902-023-01450-y)
Supplement: Supplementary file 2 — Supplementary Material 2 [file 12902_2023_1450_MOESM2_ESM.docx]

| **Table S2.** Reasons for exclusions | | | |
| --- | --- | --- | --- |
| 1. | Martin-du Pan_1998^1^ | Popolation | Control groups were patients with Hashimoto's thyroiditis or thyroid nodules |
| 2. | Chiovato_1996^2^ | Design | Incorrect type of study, literature review |
| 3. | Bagnasco_2007^3^ | Design | Incorrect type of study, literature review |
| 4. | Vos_2009^4^ | Popolation | Absence of control group |
| 5. | Paunkovic_1998^5^ | Popolation, Intervention | Absence of control group, assessment tools for stress |
| 6. | Jarusaitiene_2016^6^ | Popolation | Population with Graves’ Ophthalmopathy |
| 7. | Kahaly_2005^7^ | Popolation | Population with Graves’ Ophthalmopathy |
| 8. | Vita_2008^8^ | Design | Single case report |
| 9. | Vita_2019^9^ | Design | Single case report |
| 10. | Harris_1992^10^ | Design | Incorrect type of study, comment |
| 11. | Chiovato_1998^11^ | Intervention | Absence of SLEs assessment |
| 12. | Najafipour_2021^12^ | Popolation | Control group was also patients diagnosed with Graves’ disease |
| 13. | Williams_2022^13^ | Design | Single case report |
| 14. | Falgarone_2013^14^ | Design | Incorrect type of study, literature review |
| 15. | Stern_1996^15^ | Design | Absence of control group |
| 16. | Lee_2003^16^ | Popolation | Population with hyperthyroidism |

1. Martin-du Pan RC. Triggering role of emotional stress and childbirth. Unexpected occurrence of Graves' disease compared to 96 cases of Hashimoto thyroiditis and 97 cases of thyroid nodules. Annales D'endocrinologie. 1998. 59: 107-112.
2. Chiovato L, Pinchera A. Stressful life events and Graves' disease. European Journal of Endocrinology. 1996;134:680–2.
3. Bagnasco M, Bossert I, Pesce G. Stress and autoimmune thyroid diseases. Neuroimmunomodulation. 2006;13:309–17.
4. Vos XG, Smit N, Endert E, Brosschot JF, Tijssen JG, Wiersinga WM. Age and stress as determinants of the severity of hyperthyroidism caused by Graves' disease in newly diagnosed patients. European Journal of Endocrinology. 2009;160:193–9.
5. Paunkoviv N, Paunkovic J, Pavlovic O, Paunovic Z. The significant increase in incidence of graves' disease in eastern Serbia during the Civil War in the former Yugoslavia (1992 to 1995). Thyroid. 1998;8:37–41.
6. Jarusaitiene D, Verkauskiene R, Jasinskas V, Jankauskiene J. Predictive factors of development of graves’ ophthalmopathy for patients with juvenile graves’ disease. International Journal of Endocrinology. 2016;2016:1–9.
7. Kahaly GJ, Petrak F, Hardt J, Pitz S, Egle UT. Psychosocial morbidity of graves' orbitopathy. Clinical Endocrinology. 2005;63:395–402.
8. Vita R, Lapa D, Vita G, Trimarchi F, Benvenga S. A patient with stress-related onset and exacerbations of graves disease. Nature Clinical Practice Endocrinology & Metabolism. 2008;5:55–61.
9. Vita R, Cernaro V, Benvenga S. Stress-induced hashitoxicosis: Case report and relative HLA serotype and genotype. Revista da Associação Médica Brasileira. 2019;65:830–3.
10. Harris T, Creed F, Brugha TS. Stressful life events and Graves' disease. British Journal of Psychiatry. 1992;161:535–41.
11. Chiovato L, Marinò M, Perugi G, Fiore E, Montanelli L, Lapi P, et al. Chronic recurrent stress due to panic disorder does not precipitate graves’ disease. Journal of Endocrinological Investigation. 1998;21:758–64.
12. Najafipour M, Zareizadeh M, Najafipour F. Clinical and biochemical aspects between "stress" and "non-stress" Induced graves’ disease. Immunopathologia Persa. 2020;7.
13. Williams OC, Abdulrahim M, Davis V, Jenson C, Anand A, Bachu AK. Management of psychosis associated with graves’ disease: A rare case report. Case Reports in Psychiatry. 2022;2022:1–4.
14. Falgarone G, Heshmati HM, Cohen R, Reach G. Mechanisms in endocrinology: Role of emotional stress in the pathophysiology of graves' disease. European Journal of Endocrinology. 2013;168.
15. Stern RA, Robinson B, Thorner AR. A survey study of neuropsychiatric complaints in patients with graves' disease. The Journal of Neuropsychiatry and Clinical Neurosciences. 1996;8:181–5.
16. Lee I-T, Sheu WH-H, Liau Y-J, Lin S-Y, Lee W-J, Lin C-C. Relationship of stressful life events, anxiety and depression to hyperthyroidism in an Asian population. Hormone Research in Paediatrics. 2003;60:247–51.
