# Supplementary material for: What is the impact of stress on the onset and anti-thyroid drug therapy in patients with graves’ disease: a systematic review and meta-analysis
Source: BMC Endocr Disord. 2023 Sep 12;23:194. doi: 10.1186/s12902-023-01450-y (PMC10496195; doi:10.1186/s12902-023-01450-y)
Supplement: Supplementary file 3 — Supplementary Material 3 [file 12902_2023_1450_MOESM3_ESM.docx]

| **Table S3.** General information and features of the included 13 studies | | | | | | |
| --- | --- | --- | --- | --- | --- | --- |
| **Study ID** | 1 | **Study DOI** | 10.1093/oxfordjournals.qjmed.a067837 | | | |
| **Study Title** | Thyrotoxicosis and Stress | | | | | |
| **First Author** | Gray J | **Year** | 1985 | **Country** | UK | |
| **Continent** | Europe | **Patients, N** | 50 | **Controls, N** | 50 | |
| **Instrument** | SSI | **Comparator** | Control | **Outcome** | Graves’ disease | |
| **Design** | Case-control | **Ref. number** | 15 | **Source** | Journal article | |
| **Study ID** | 2 | **Study DOI** | 10.1016/0140-6736(91)92298-G | | | |
| **Study Title** | Stressful life events and Graves' disease | | | | | |
| **First Author** | Winsa B | **Year** | 1991 | **Country** | Sweden | |
| **Continent** | Europe | **Patients, N** | 208 | **Controls, N** | 372 | |
| **Instrument** | LES | **Comparator** | Control | **Outcome** | Graves’ disease | |
| **Design** | Case-control | **Ref. number** | 11 | **Source** | Journal article | |
| **Study ID** | 3 | **Study DOI** | 10.1530/acta.0.1280293 | | | |
| **Study Title** | Life events in the pathogenesis of Graves' disease. A controlled study | | | | | |
| **First Author** | Sonino N | **Year** | 1993 | **Country** | | Italy |
| **Continent** | Europe | **Patients, N** | 70 | **Controls, N** | | 70 |
| **Instrument** | PIRLE | **Comparator** | Control | **Outcome** | | Graves’ disease |
| **Design** | Case-control | **Ref. number** | 12 | **Source** | | Journal article |
| **Study ID** | 4 | **Study DOI** | 10.1111/j.1365-2265.1995.tb01879.x | | | |
| **Study Title** | Life events, daily stresses and coping in patients with Graves' disease | | | | | |
| **First Author** | Kung A | **Year** | 1995 | **Country** | | China |
| **Continent** | Asia | **Patients, N** | 95 | **Controls, N** | | 95 |
| **Instrument** | LES | **Comparator** | Control | **Outcome** | | Graves’ disease |
| **Design** | Case-control | **Ref. number** | 13 | **Source** | | Journal article |
| **Study ID** | 5 | **Study DOI** | 10.1530/eje.0.1340699 | | | |
| **Study Title** | Stressful life events in the pathogenesis of Graves’ disease | | | | | |
| **First Author** | Radosavljević | **Year** | 1996 | **Country** | | Serbia |
| **Continent** | Europe | **Patients, N** | 100 | **Controls, N** | | 100 |
| **Instrument** | PIRLE | **Comparator** | Control | **Outcome** | | Graves’ disease |
| **Design** | Case-control | **Ref. number** | 14 | **Source** | | Journal article |
| **Study ID** | 6 | **Study DOI** | 10.1097/00006842-199803000-00013 | | | |
| **Study Title** | Stressful life events and smoking were associated with Graves' disease in women, but not in men | | | | | |
| **First Author** | Yoshiuchi K | **Year** | 1998 | **Country** | | Japan |
| **Continent** | Asia | **Patients, N** | 228 | **Controls, N** | | 228 |
| **Instrument** | HRLES | **Comparator** | Control | **Outcome** | | Graves’ disease |
| **Design** | Case-control | **Ref. number** | 16 | **Source** | | Journal article |
| **Study ID** | 7 | **Study DOI** | 10.1046/j.1365-2265.2001.01332.x | | | |
| **Study Title** | Relationship between the number and impact of stressful life events and the onset of Graves' disease and toxic nodular goitre | | | | | |
| **First Author** | Matos-Santos A | **Year** | 2001 | **Country** | | Portugal |
| **Continent** | Europe | **Patients, N** | 31 | **Controls, N** | | 31 |
| **Instrument** | LES | **Comparator** | Control | **Outcome** | | Graves’ disease |
| **Design** | Case-control | **Ref. number** | 23 | **Source** | | Journal article |

| **Table S3.** (continued) | | | | | | |
| --- | --- | --- | --- | --- | --- | --- |
| **Study ID** | 8 | **Study DOI** | NA | | | |
| **Study Title** | Stressful life events and Graves' disease: Results of a case control study | | | | | |
| **First Author** | Pintor A | **Year** | 2003 | **Country** | Philippines | |
| **Continent** | Asia | **Patients, N** | 224 | **Controls, N** | 224 | |
| **Instrument** | LES | **Comparator** | Control | **Outcome** | Graves’ disease | |
| **Design** | Case-control | **Ref. number** | 24 | **Source** | Journal article | |
| **Study ID** | 9 | **Study DOI** | 10.3109/13651501.2011.631016 | | | |
| **Study Title** | Effect of stressful life events on the initiation of graves’ disease | | | | | |
| **First Author** | Topcu C | **Year** | 2012 | **Country** | Turkey | |
| **Continent** | Asia, Europe | **Patients, N** | 45 | **Controls, N** | 37 | |
| **Instrument** | LES | **Comparator** | Control | **Outcome** | Graves’ disease | |
| **Design** | Case-control | **Ref. number** | 25 | **Source** | Journal article | |
| **Study ID** | 10 | **Study DOI** | 10.1097/00006842-199809000-00014 | | | |
| **Study Title** | Psychosocial factors influencing the short-term outcome of antithyroid drug therapy in Graves' disease | | | | | |
| **First Author** | Yoshiuchi K | **Year** | 1998 | **Country** | | Japan |
| **Continent** | Asia | **Cured cases, N** | 155 | **Noncured cases, N** | | 75 |
| **Instrument** | HRLES | **Comparator** | Not-cured, or recurrences | **Outcome** | | Drug efficacy |
| **Design** | Prospective | **Ref. number** | 26 | **Source** | | Journal article |
| **Study ID** | 11 | **Study DOI** | 10.1046/j.1365-2265.2003.01625.x | | | |
| **Study Title** | The relationship of psychological factors to the prognosis of hyperthyroidism in antithyroid drug-treated patients with Graves’ disease | | | | | |
| **First Author** | Fukao A | **Year** | 2003 | **Country** | | Japan |
| **Continent** | Asia | **Cured cases, N** | 41 | **Noncured cases, N** | | 28 |
| **Instrument** | NSI | **Comparator** | Not-cured, or recurrences | **Outcome** | | Drug efficacy |
| **Design** | Prospective | **Ref. number** | 27 | **Source** | | Journal article |
| **Study ID** | 12 | **Study DOI** | 10.1055/s-0032-1323807 | | | |
| **Study Title** | Mental Health Status and Factors that Influence the Course of Graves’ Disease and Antithyroid Treatments | | | | | |
| **First Author** | Chen D | **Year** | 2012 | **Country** | | China |
| **Continent** | Asia | **Cured cases, N** | 129 | **Noncured cases, N** | | 148 |
| **Instrument** | LES | **Comparator** | Not-cured, or recurrences | **Outcome** | | Drug efficacy |
| **Design** | Prospective | **Ref. number** | 28 | **Source** | | Journal article |
| **Study ID** | 13 | **Study DOI** | 10.1007/s12020-014-0289-8 | | | |
| **Study Title** | Stress triggers the onset and the recurrences of hyperthyroidism in patients with Graves' disease | | | | | |
| **First Author** | Vita R | **Year** | 2014 | **Country** | | Italy |

| **Table S3.** (continued) | | | | | |
| --- | --- | --- | --- | --- | --- |
| **Continent** | Europe | **Cured cases, N** | 43 | **Noncured cases, N** | 15 |
| **Instrument** | SSI | **Comparator** | Not-cured, or recurrences | **Outcome** | Drug efficacy |
| **Design** | Prospective | **Ref. number** | 29 | **Source** | Journal article |
| Abbreviations: SSI, Semi-structured interview; Ref, Reference; NA, Not available; LES, The Life Experiences Survey; PIRLE, Paykel's Interview for Recent Life Events; HRLES, the Holmes and Rahe life events scale; NSI, the Natsume’s Stress Inventory. | | | | | |
